# Size Distribution and Metal Concentrations of Particulate Matter (PM) Sourced from Fireworks Shows in the Indoor Air of Wedding Halls in Bursa, Türkiye

**DOI:** 10.3390/toxics14050377

**Published:** 2026-04-28

**Authors:** Mert Karacalı, Burcu Onat, Ülkü Alver Şahin, Sabahattin Sıddık Cindoruk, Berna Kırıl Mert

**Affiliations:** 1 Environmental Protection and Control Department, Bursa Metropolitan Municipality, 16080 Bursa, Türkiye; 2 Environmental Engineering Department, Engineering Faculty, Istanbul University-Cerrahpaşa, 34315 Istanbul, Türkiye; bonat@iuc.edu.tr (B.O.); ulkualver@iuc.edu.tr (Ü.A.Ş.); 3 Department of Environmental Engineering, Faculty of Engineering, Bursa Uludag University, 16059 Bursa, Türkiye; cindoruk@uludag.edu.tr; 4 Department of Environmental Engineering, Faculty of Engineering, Sakarya University, 54187 Sakarya, Türkiye

**Keywords:** indoor air quality, particle exposure, particle size distribution, metal composition, fireworks

## Abstract

Fireworks are widely used at festive events worldwide and are commonly employed at indoor wedding ceremonies in Türkiye; however, their impact on indoor air quality has not been adequately investigated. This study examines particulate matter (PM) emissions generated by volcano-type fireworks used in indoor wedding halls. Particle samples were collected across five size fractions (>2.5, 1.0–2.5, 0.50–1.0, 0.25–0.50, and <0.25 µm) using a 5-stage Sioutas cascade impactor, and elemental compositions were determined by ICP-MS. PM_2.5_ concentrations ranged between 1518 and 7796 µg/m^3^, while total PM concentrations varied between 2088 and 8656 µg/m^3^. These values are substantially higher than the guideline limits reported by the World Health Organization (WHO) and the United States Environmental Protection Agency (USEPA), indicating very high short-term exposure levels. Fine particles (PM_2.5_) accounted for 56.5–92.4% of total PM, with particles smaller than 1 µm forming the dominant fraction. Among the analyzed elements, aluminum (Al), zinc (Zn), and iron (Fe) were the most abundant metals, predominantly associated with fine particles. The highest concentrations were generally observed in the 0.25–0.5 µm size range. These findings showed that the use of indoor fireworks can lead to high concentrations of fine particulate matter and metal-rich aerosols. This issue should be considered, as it may pose a health risk for those in enclosed spaces in future studies.

## 1. Introduction

Air pollution is one of the most pressing environmental problems worldwide, directly associated with adverse effects on human health and ecosystems. It contributes to increased morbidity and mortality, particularly in densely populated urban areas. Current estimates suggest that more than one billion people live in regions with air quality below acceptable levels [1,2]. The U.S. Environmental Protection Agency (EPA) defines air pollution as the presence of pollutants in the atmosphere that can negatively impact human health, welfare, or the environment [3].

In addition to outdoor pollution, indoor air pollution has emerged as a major concern, as individuals spend most of their time in enclosed spaces. Indoor pollution refers to contaminants in non-industrial buildings, such as homes, offices, schools, and public facilities. The World Health Organization (WHO) has reported that indoor air pollution accounts for 2.7% of the global burden of disease [4]. Pollutants such as particulate matter (PM), nitrogen oxides (Nox), carbon monoxide (CO), carbon dioxide (CO_2_), volatile organic compounds (VOCs), polycyclic aromatic hydrocarbons (PAHs), formaldehyde, radon, cigarette smoke, allergens, and mineral fibers have been identified as key contributors to poor indoor air quality [5,6,7,8,9,10,11,12]. Since indoor air quality is strongly linked to outdoor conditions, infiltration of outdoor pollutants further exacerbates exposure risks.

Particulate matter is considered one of the most hazardous pollutants due to its small size, chemical composition, and ability to penetrate deep into the respiratory system. PM_10_ includes particles such as dust and pollen, while PM_2.5_ consists of finer particles, including combustion by-products and organic compounds. Both fractions are associated with significant health effects, ranging from respiratory ailments to carcinogenic outcomes [13,14]. Indoor PM levels vary with ventilation, building materials, and human activities, making indoor exposure particularly complex [15,16,17]. WHO emphasizes the “Rule of 1000,” which highlights that pollutants generated indoors are far more likely to be inhaled than those released outdoors. Each year, 3.8 million deaths are attributed to indoor air pollution, compared with 4.2 million from outdoor pollution [18,19]. These findings emphasize that strategies to improve air quality must focus not only on outdoor environments but also on indoor settings where exposure risks may be higher.

Although some air pollution sources are natural, such as forest fires, volcanic activity, desert dust, or lightning, anthropogenic emissions are more widespread and cause greater environmental and health problems [20]. Among these, fireworks represent a significant yet often overlooked short-term source of pollution [21]. Traditionally used in festivals and celebrations, fireworks are especially common in Turkey during weddings and cultural events [22].

Several studies have shown that during and immediately after firework displays, air pollutant concentrations increase dramatically, often between two and eight times higher than background levels before gradually returning to normal within about 24 h. The smoke produced by fireworks can reduce visibility by over 90%. Their combustion releases fine particles enriched with metals such as magnesium (Mg), aluminum (Al), iron (Fe), and zinc (Zn), as well as water-soluble ions including potassium (K^+^), chloride (Cl^−^), and sulfate (SO_4_^2−^). These phenomena have been documented during various celebrations, including the Sant Joan festival in Spain, the World Cup celebrations in Italy, the Festival of Lights in India, the Lantern Festival in Taiwan, and the Spring Festival in China [23].

The chemical composition of fireworks is central to their environmental impact. They typically contain oxidizers (such as nitrates, chlorates, and perchlorates), fuels (aluminum, magnesium, or coal), and coloring agents (strontium and barium salts) [24]. When ignited, these components generate particulate matter, carbon monoxide, nitrogen oxides, sulfur dioxide, ozone, and other toxic by-products. The released particles often contain toxic metals such as cadmium (Cd), copper (Cu), lead (Pb), chromium (Cr), manganese (Mn), and barium (Ba), which can accumulate in the environment and enter the food chain [25,26,27,28]. Health studies have demonstrated that exposure to these metals may cause neurotoxic effects, respiratory impairment, and increased risks of cancer [29]. Fine and ultrafine fractions of fireworks-related PM are particularly dangerous, as they can penetrate deeply into the lungs and bloodstream, aggravating conditions such as asthma and cardiovascular disease [30,31,32].

Despite growing international awareness of the environmental and health risks associated with fireworks, research on their effects in indoor environments remains limited. This gap is especially important in Turkey, where fireworks are frequently used in enclosed wedding halls, potentially exposing large groups of people to elevated pollutant concentrations in confined spaces. However, little is known about the specific levels of particulate matter and associated metals released indoors, or how these pollutants may impact human health under such conditions.

To address this knowledge gap, the present study focuses on the use of volcano-type fireworks in wedding halls in Bursa, Turkey. By sampling particulate matter in indoor air during these events, the study evaluates size distribution, concentration, and chemical composition, aiming to provide new insights into the health risks posed by indoor fireworks emissions.

## 2. Materials and Methods

### 2.1. Sampling and Gravimetric Analysis

Bursa, in northwest Turkey, lies southeast of the Sea of Marmara (40°15′–41°38′ N, 28°40′–30°48′ E) with a population of about 3 million. Key industries include textile, automotive, metal, food, and leather [33].

In 10 wedding halls in Bursa, samples were collected from indoor air during the explosion of volcano-type fireworks to determine the particle size distribution and metal content of the particulate matter. All materials used in the study were indoor pyrotechnic products manufactured by Venüs Fireworks (Sakarya, Turkey). The technical specifications of the pyrotechnic materials analyzed in this study were standardized to ensure the reproducibility of the experiments. All sampled volcano-type fireworks were 3 inches in size and, under controlled conditions, exhibited an average burning time of 30 s and a smoke rise height of 3 m. Model-based details and the quantities of the materials used in the experiments are presented in Table 1.

**Figure 1:**
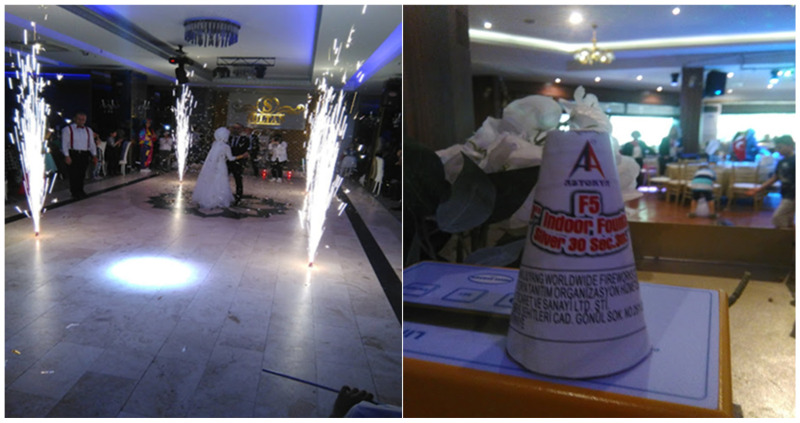
Fireworks Show in Wedding Halls and Firework Type.

The sampling process was carried out from an average distance of 3–5 m from the exploding material and from a height of 1.5 m above the ground. The sampling process began 10 min before the fireworks were set off. The sampling process lasted 60 min after the explosion. Images of volcano-type fireworks exploding in wedding halls are given in Figure 1.

Indoor airborne particle sampling was performed using a 5-stage Sioutas personal cascade impactor (Sioutas Cascade Impactor, model SKC) and a 9 L/min sampling pump (SKC Leland Legacy, Dorset, UK, origin is USA). The particle size ranges collected on the filter were >2.5 µm, 1.0–2.5 µm, 0.50–1.0 µm, 0.25–0.50 µm, and <0.25 µm. Particles larger than 0.25 µm were collected in a 25 mm PTFE filter, and particles smaller than 0.25 µm were collected in a 37 mm PTFE filter. The SKC Leland Legacy pump was calibrated with a DryCal calibrator (Bios-IC, Lakewood, NJ, USA). Gravimetric analysis was performed with a precision electronic balance with an accuracy of 0.01 mg. Filters were conditioned at 20 ± 2 °C and 50 ± 5% relative humidity before and after sampling.

### 2.2. Extraction and Metal Analysis

Various analytical methods, including atomic absorption spectrometry (AAS), inductively coupled plasma optical emission spectrometry (ICP-OES), and inductively coupled plasma mass spectrometry (ICP-MS), have been used to detect heavy metal elements. Among these techniques, ICP-MS is the most common due to its advantages, including extremely high sensitivity, a wide dynamic range, and the ability to analyze multiple elements simultaneously [34].

Inductively Coupled Plasma Mass Spectrometry (ICP-MS) (Perkin Elmer Model) was conducted to analyze metals in the PM. The filters were digested using a Microwave Digestion System (MARS 5) to prepare a solution for metal analysis. Acid dissolution of filtres was performed with 5 mL of 65% nitric acid (HNO_3_), 1 mL of 30% hydrochloric acid (HCl), and 0.5 mL of hydrogen fluoride (HF) acid. Digestion was performed simultaneously on a blank filter, five sample filters, and a control solution. After digestion, the sample solutions were directly diluted to 15 mL with deionized distilled water and stored in the refrigerator at approximately −4 °C before analysis. The measurement was considered accurate when the repeatability of the three measurements for each sample was less than 5%. Fe, Ti, Sn, Al, Sr, Sb, and Zn, elements commonly found in fireworks, were analyzed by ICP-MS [35,36]. The limit of detection (LOD) of metal by ICP-MS is given in Table S1 in the Supplementary Materials.

## 3. Results and Discussion

### 3.1. Particulate Matter Concentration and Size Distribution

The mass concentrations of PM collected on filters of different sizes in wedding halls were calculated using the gravimetric method, and the results are given in Table 2 (also Table S2). It was found that PM > 2.5 (coarse particles) concentrations ranged from 567 µg/m3 to 1574 µg/m3, and PM2.5 (fine particles) concentrations ranged from 1518 µg/m3 to 7796 µg/m^3^. The highest concentrations of PM_2.5_ were determined as 7796 µg/m^3^ and 6939 µg/m^3^ in Hall 3 and Hall 4, respectively. It was observed that the PM_2.5_ fraction of total PM ranged from 56.5% to 92.4%, with fine particles being the dominant component.

There is no limit value for PM_2.5_ in our country yet; the United States Environmental Protection Agency (USEPA) has accepted the 24 h averOrganizationandard of 35 µg/m^3^ [37]. The World Health Organization (WHO) recommends a limit value of 15 µg/m3 for PM2.5 [19]. In our country, the limit value for the 8 h average respirable dust (<5 microns) is 5 mg/m^3^, and the limit value for total dust is 15 mg/m^3^, according to the Occupational Health and Safety Regulation on Dust Control. This study found that the average PM_2.5_ concentrations in all wedding halls were well above the EPA standard and the WHO-recommended limit. Compared to the limit value given in the Regulation on Combating Dust, the average PM concentration in Hall 3 and Hall 4 exceeded the limit value of 5 mg/m_3_ for respirable dust. However, the average concentrations determined in this study represent a sampling period of approximately 70 min. Given the 8 h exposure time, the average PM concentration across all halls will remain below the limit of 5000 µg/m^3^.

**Figure 2:**
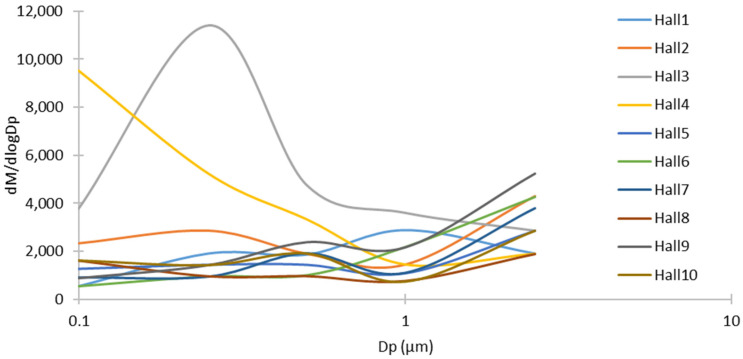
Particle mass size distribution for all wedding halls.

Figure 2 shows the particle size distributions of total PM sampled in 10 wedding halls. It was observed that the main component of total PM in all halls was particles smaller than 1 μm, and the particle size distribution was similar in all halls except Hall 3 and Hall 4. In Halls 3 and 4, particles smaller than 0.5 μm are more abundant. Differences in the production and use of fireworks may alter particle size distributions. These results are similar to those reported by Salma et al. during fireworks displays in Budapest, Hungary [38]. The study conducted in Budapest found that the average PM_10_ concentration over 1 h increased by 123 times compared to the concentration before the demonstration, and the highest particle diameter range was 0.1–1 μm.

### 3.2. Metal Concentration

The concentrations of Fe, Ti, Sn, Al, Sr, Sb, and Zn in PM sampled in five different sizes in wedding halls were determined by ICP-MS analysis. The average concentrations in total PM were determined as 403.77 μg/m^3^ for Al, 240.96 μg/m^3^ for Zn, 209.96 μg/m^3^ for Fe, 5.37 μg/m^3^ for Sr, 5.32 μg/m^3^ for Sn, 0.477 μg/m^3^ for Sb, and 0.0078 μg/m^3^ for Ti (Table S3). Al was the metal with the highest concentration in all samples. Al is one of the most widely used components in explosive and flammable products due to bright flames and white sparks [39]. In the measurement results, Hall 6 has the highest Al concentration (0.5–1.0 μm, 1596.53 μg/m^3^), while Hall 4, Hall 5, Hall 7, and Hall 8 have the highest Al concentrations. In Halls 1 and 2, the lowest Al level was analyzed. In the PM > 2.5 fraction, Al levels are generally low, but in some halls, such as Hall 5 and Hall 9, they are quite high. In the PM fraction < 0.25, an increase in Al levels is generally observed. In general, as the PM decreases, Al concentration increases.

Zn and Fe have the second- and third-highest concentrations, respectively, after Al. Zn is widely used to produce smoke effects in fireworks and pyrotechnics. Al and Zn are typically used instead of Mg in pyrotechnic compositions to decrease the melting point and ignition temperature of the flash mixtures [40].

In our study, Hall 4 has the highest Zn content (375.41 μg/m^3^) in the 0.25–0.5 μm PM size range. It was determined that the Zn parameter reached high levels in Hall 3 at sizes of 0.5–1.0 μm and PM < 0.25, and was at the lowest levels in Hall 1 and Hall 2. Zn concentrations increase significantly as PM gets smaller.

Fe is widely used for forming fuel and golden sparks [36]. Hall 6 had the highest Fe concentration levels of 87.95 μg/m^3^ at 0.5–1.0 μm particle size, while a significant decrease in Fe was observed for PM < 0.25 μm. Halls 5, 7, 8, and 9 had high Fe levels (50–80 μg/m^3^ range), while the lowest Fe levels were around 0.1 μg/m^3^ in Hall 3 (PM > 2.5) and Hall 10 (PM > 2.5). Fe levels fluctuated as PM decreased, and in some halls, Fe was concentrated in large particle sizes, whereas it was detected more in medium particle sizes. Kulshrestha et al. [41] conducted an indoor air assessment without fireworks in 2014. When the concentrations of individual metals in PM_10_ and PM_2.5_ were analyzed indoors in roadside, rural, and urban settlements, the trends were Fe > Zn > Pb > Cr > Ni > Cu > Mn [41].

In addition to Al, Fe, and Zn, components such as Ti, Sr(NO_3_)_2_, SnO, and SnO_2_ are used in volcano-type fireworks preferred in indoor environments. In this study, Sn and Sr were detected in the samples, and Ti and Sb showed lower concentrations. The low concentrations of Sb and Ti indicate that they are minor components of the explosive. These metals were likely converted to various oxides during pyrotechnic combustion (Saporito et al. [32]. The highest Sb concentration levels (0.5–1.0 μm, 0.228 μg/m^3^) were detected in Hall 6, while Hall 5 and 7 also had high Sb levels (0.145–0.170 μg/m^3^). As with other metals, the lowest Sb levels were observed in Halls 1 and 3. Sb could not be measured in some PM fractions of Hall 1. Sb levels appear to fluctuate slightly as PM size decreases, but do not show significant changes. In general, Ti concentrations ranged from 0 to 0.01 μg/m^3^, and Ti levels were generally very low; the highest Ti value was 0.01 μg/m^3^ for PM > 2.5 in Halls 5 & 9. In most halls, the Ti concentration did not change significantly as the PM size decreased.

SnO and SnO_2_ are used as active components in fireworks [42]. The highest Sn concentration values were 0.25–0.5 μm and 2.32 μg/m^3^ for Hall 9. Measurements were made at the levels of (PM < 0.25, 2.29 μg/m^3^), (PM < 0.25, 1.115 μg/m^3^), PM < 0.25, 1.555 μg/m^3^), (PM < 0.25, 1.375 μg/m^3^), (PM > 2.5, 1.315 μg/m^3^), 0.5–1.0 μm, 1.355 μg/m^3^) in Halls 2, 4, 5, 6, 7, and 8, respectively. The lowest Sn values were observed in Hall 1 (PM < 0.25, 0.01 μg/m^3^), and Sn levels are generally lower in the PM > 2.5 fraction, except in Hall 3 (2 μg/m^3^). In the PM fraction < 0.25, Sn levels generally increase. This distribution shows that the Sn parameter is generally more concentrated as particle size decreases (in small-size fractions such as PM < 0.25).

Sr(NO_3_)_2_ is rarely used as an oxidizer in fireworks. Sb_2_S_3_ is often used in the composition of fountain-type fireworks to produce bright red, blue, and purple light [32]. Hall 6 has high Sr components, as in the Al metal parameter (0.5–1.0 μm, 2.09 μg/m^3^). Hall 6 and Hall 9 have high Sr levels. Halls 1, 2, and 3 have the lowest Sr levels. Especially in Hall 1, Sr could not be measured in the PM_2.5_ fraction. Sr metal level generally increased as PM decreased.

**Figure 3:**
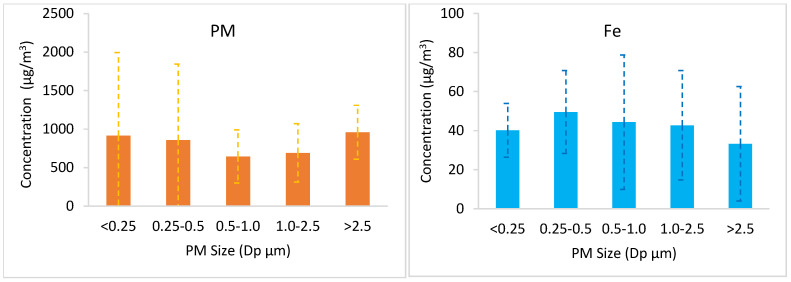

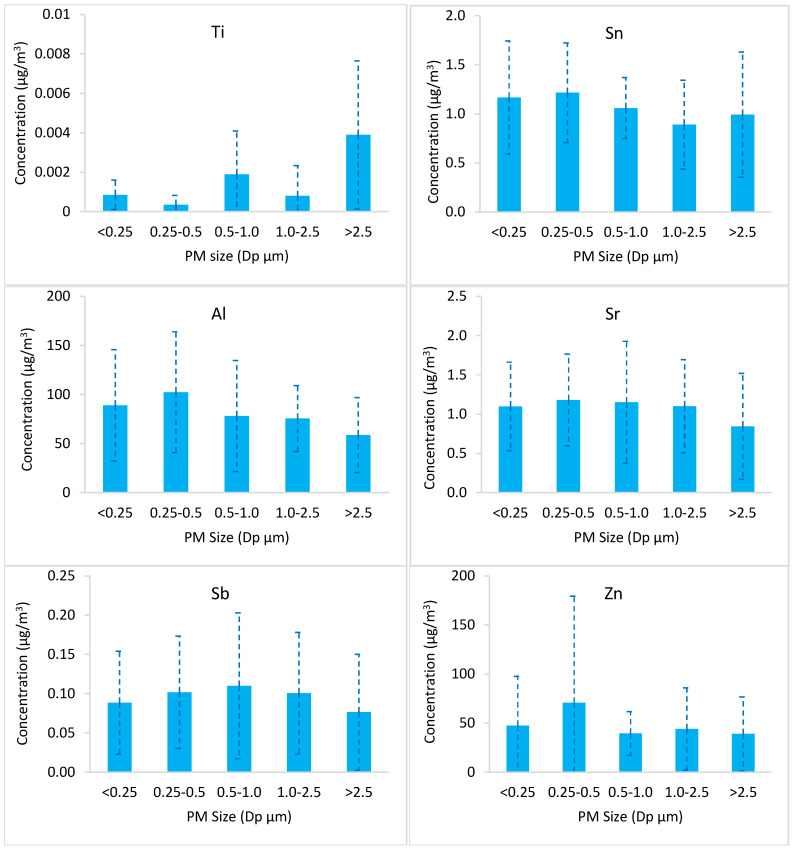
Average PM and metal concentrations in five different size fractions.

Particle size distributions of the analyzed metals and PM are given in Figure 3. In general, it was observed that all metals except Ti showed a balanced distribution similar to the size distribution of PM in 5 different particle fractions. It was determined that the particle size range with the highest concentration of Fe, Sn, Sr, Zn, and Al was 0.25–0.5 µm, and the particle size range with the highest concentration of Sb was 0.5–1.0 µm. It was observed that all metals except Ti were collected in PM_2.5_, and PM_2.5_ concentrations were higher than those of PM > 2.5.

When the distribution of Ti concentration across particle sizes is examined, it is found that Ti is concentrated in particles larger than 2.5 µm, unlike other elements. This is because the silver-coloured sparkle image in volcano-type fireworks is produced by burning Ti metal in the form of powder or particles [38].

In an indoor study similar to this one, the concentrations of heavy metals (Ag^+^, Cd^2+^, Cr^2+^, and Pb^2+^) were investigated to assess their risk to human health. Samples were collected using a high-volume air sampler installed after silver fireworks were lit in a laboratory, where indoor conditions were simulated by keeping windows and doors closed. The results showed that the concentrations exceeded the permissible values of the US National Ambient Air Quality Standards (0.0005, 0.004, and 0.6 μg/cm^3^) for rural, urban, and industrial areas. Higher concentrations of Ag^+^, Cd^2+^, and Cr^2+^ indicated greater toxicity. The evaluation of the potential health risks posed by these metals showed they were highly harmful [43].

**Figure 4:**
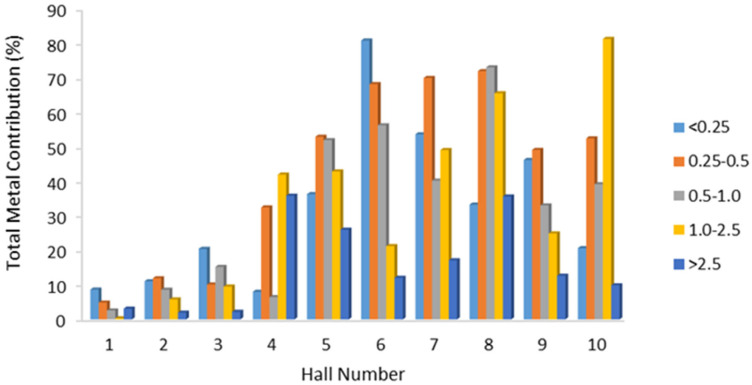
Percentage distribution of total metal ratios in different PM sizes.

In Figure 4, the total metal ratios in PM sampled in 5 different sizes are given as percentages. The metal ratios in PM_2.5_ are high in all halls except halls 1, 2, and 3. In hall 6, approximately 80% of particles smaller than 0.25 were composed of metals. Similarly, in halls 5, 6, 7, 8, 9, and 10, the ratio of metals in particles of 0.25–0.5 microns ranges from 48% to 70%. The color, type, and intensity of fireworks are considered the reasons for the differences in metal content [44,45,46].

The United States Environmental Protection Agency (USEPA) identified three exposure routes in the risk assessment: inhalation, ingestion, and dermal contact [37,47]. Inhalation is the most rapid exposure pathway. Generally, an exposure pathway defines the process by which a stressor can come into contact with receptors. Health risks posed by contaminated metals were assessed by calculating hazard quotients for ingestion and dermal absorption, revealing the combined potential health risks to humans exposed to different heavy metals. Risks include cuts, burns, fatigue, and fumes. Major health implications include kidney damage, degenerative neurological conditions, cancer, respiratory and cardiovascular diseases [43].

The fact that a significant proportion of the metals detected in samples taken during fireworks in wedding halls are present in particles smaller than 2.5 µm requires that these particles be considered for their potential adverse health effects when inhaled. There is no regulation on indoor air quality in our country yet, nor any limit values for these metals in the rules on outdoor air quality. According to Occupational Health and Safety regulations, the Regulation on Health and Safety Measures in Working with Chemical Substances and the U.S. Occupational Safety and Health Administration (OSHA) set the TWA (time-weighted average) limits for Al metal at 15 mg/m^3^ for total dust and 5 mg/m^3^ for respirable dust over 8 h. In OSHA, the TWA value for Sn metal in total dust is given as 2 mg/m^3^ (inorganic compound) and 0.1 mg/m^3^ (organic compound), and the TWA value for Sb metal in total dust is 0.5 mg/m^3^. In this study, Al concentrations detected in total dust were below the 15 mg/m^3^ limit. This study found that Sn and Sb concentrations in total dust were below the OSHA limit values [48,49].

In addition to the statistical analysis, the relationships between particle size fractions and metal concentrations were qualitatively examined. The results indicate that most metals, particularly Al, Zn, and Fe, are associated with fine particles, with a distribution pattern consistent with the overall PM size distribution. Higher metal concentrations were generally observed in smaller particle fractions, especially within the 0.25–0.5 µm range.

However, due to the limited number of samples per wedding hall, robust statistical correlations could not be established with sufficient confidence. Therefore, the observed relationships are interpreted as general trends rather than definitive quantitative associations. These findings are nevertheless consistent with previous studies and support the conclusion that fine particles play a key role in transporting metal pollutants generated by fireworks.

### 3.3. Result of Statistical Analysis

We added a dedicated Statistical Analysis subsection and provided descriptive statistics (mean, standard deviation, min–max) for each metal across size fractions (Table S3).

### 3.4. Short-Term Exposure and Risk Context

Their combustion produces smoke, noise, and visual displays, but also releases pollutants into the atmosphere. Fireworks emissions include particulate matter, gases, and heavy metals that adversely affect local air quality and visibility [21]. Beyond direct hazards such as accidental detonations or hearing damage from high sound pressure levels, the inhalation of pollutants generated during fireworks displays represents a serious risk for both humans and the environment [50,51,52,53,54].

We estimated short-term inhalation exposure for attendees, assuming 1 h of light activity (Inhalation rate = 1.2 m^3^/h). Based on measured PM2.5 (Aver = 7.71 mg/m^3^; Max = 17.14 mg/m^3^), the inhaled PM_2.5_ mass is ≈ 9.25 mg (mean) and ≈ 20.57 mg (peak hall). For metals in PM_2.5_, the average inhaled mass over 1 h is ≈ 2.07 mg Al, ≈ 1.21 mg Zn, and ≈ 0.42 mg Fe. We provide a qualitative risk context against the WHO PM_2.5_ guidelines and OSHA metal limits and discuss that acute peaks of ultrafine/fine particles carry heightened respiratory risk despite short durations.

Although the Incremental Lifetime Cancer Risk (ILCR) is a widely used indicator for long-term health risk assessment, it was not calculated in this study because the focus was on short-term exposure conditions during firework events. ILCR assessments typically require long-term exposure assumptions and chronic intake parameters, which are not directly applicable to the short-duration, event-based exposure scenario investigated in this study.

Instead, the evaluation was based on short-term inhalation exposure estimates and comparison with established guideline values, which are more appropriate for assessing acute exposure risks. Nevertheless, the inclusion of ILCR-based assessments could provide additional insight and is recommended for future studies incorporating long-term exposure data.

### 3.5. Limitations

This study has several limitations that should be considered when interpreting the findings. First, sampling was limited to single 70 min windows, which may not fully capture the temporal variability of pollutant concentrations during or after firework displays. Event-based sampling reflects realistic exposure conditions despite limited replication. Second, the absence of personal exposure monitoring limited the ability to directly assess the health risks associated with individual-level exposures. Third, the lack of real-time particulate matter instrumentation limited the capacity to observe short-term concentration peaks and dynamic changes in particle size distribution. This study primarily focused on characterizing short-term exposure during active firework events in indoor environments, aiming to capture peak pollutant concentrations under realistic conditions. Due to the event-based nature of fireworks usage and logistical constraints in operational wedding halls, time-resolved sampling at multiple post-ignition intervals (e.g., 1 h, 2 h, and 4 h) could not be conducted within the scope of this study.

Nevertheless, the high concentrations observed during the sampling period clearly indicate that fireworks represent a significant short-term pollution source in enclosed environments. Investigation of the temporal attenuation and removal dynamics of these pollutants remains an important area for future research to better understand indoor air recovery processes and exposure duration. Finally, using a single replicate per wedding hall reduced the statistical power of inferential analyses, thereby constraining the generalizability of the results. Future research incorporating longer monitoring periods, personal exposure assessments, real-time instrumentation, and multiple replicates would strengthen the robustness of conclusions regarding indoor air pollution from fireworks and its health implications. Variability in firework composition from unknown manufacturers may have influenced the particle size distribution. Background concentrations were assumed to be negligible compared to peak levels observed during fireworks events. Selected metals represent dominant pyrotechnic components rather than a full toxicological screening.

## 4. Conclusions

In this study, the particle size distribution and particle concentration of volcano-type fireworks, which exploded for demonstration purposes in 10 different wedding halls in Bursa province, were determined, and their chemical composition was analyzed. Although the burn time of volcano-type fireworks was short, the average PM_2.5_ concentration during and after the explosion, over a total exposure time of 70 min, was well above the WHO and EPA limits. It was determined that PM_2.5_ constituted 56.5% and 92.4% of the total particulate matter, especially particles smaller than 1 micron, which were the main component of total PM. The metals with the highest concentrations in total dust were Al, Fe, and Zn, and metals were mainly concentrated in fine particles. Therefore, it should be taken into consideration that the fact that the particles emitted to the environment are mainly composed of particles smaller than 1.0 µm may cause adverse health effects, even for a short time, depending on the distance to the explosion process and the type and chemical content of the fireworks, especially for children, the elderly and people with chronic respiratory diseases among the audience. For this reason, the use of such products for demonstration and show purposes in closed areas should be prevented. The results of this study were shared with the local government, and fireworks were banned in indoor venues such as wedding halls. This study is intended as a reference for countries where fireworks are widely used indoors.

## Figures and Tables

**Table 1:** Properties of Materials Used in the Sampled Volcano-Type Fireworks.

The Sampled Volcano-Type Fireworks						
Sample No.	Name	Quantity	Type	Average Burning Time (sn)	Size (inç)	Average Smoke Height (m)
1	FRK-VO2	4	Volcano-type (white, odorless)	30	3	3
2	MK-F04	4	Volcano-type (white, odorless)	30	3	3
3	MK-F04	2	Volcano-type (white, odorless)	30	3	3
4	F5 (Astorya)	2	Volcano-type	30	3	3
5	BCSP-3015	2	Volcano-type	30	3	3
6	BCSP-3015	2	Volcano-type	30	3	3
7	BCSP-3015	2	Volcano-type	30	3	3
8	BCSP-3015	2	Volcano-type	30	3	3
9	MK-F04 BCSP-3015	1 1	Volcano-type	30	3	3
10	BCSP-3015	2	Volcano-type	30	3	3

**Table 2:** Particulate matter concentrations in different size fractions, μg/m^3^.

Wedding Hall No.	A PM >2.5	B PM _1.0–2.5_	C PM _0.5–1.0_	D PM _0.25–0.5_	F PM < 0.25	PM >2.5	PM_2.5_	PM Total	% PM 2.5	% PM >2.5
1	573	1148	560	574	214	569	2497	3066	81.4	18.6
2	1290	577	572	860	928	1290	2937	4226	69.5	30.5
3	860	1434	1431	3431	1500	860	7796	8656	90.1	9.9
4	572	577	1002	1574	3786	567	6939	7507	92.4	7.6
5	860	425	429	430	500	860	1784	2644	67.5	32.5
6	1280	862	300	285	214	1280	1661	2941	56.5	43.5
7	1145	433	574	280	357	1145	1644	2789	58.9	41.1
8	570	300	288	287	643	574	1518	2088	72.7	27.3
9	1574	862	717	430	353	1574	2363	3937	60.0	40.0
10	860	291	574	428	643	860	1936	2796	69.2	30.8

## Data Availability

The original contributions presented in this study are included in the article/Supplementary Materials. Further inquiries can be directed to the corresponding author.

## Supplementary Materials

The following supporting information can be downloaded at: https://www.mdpi.com/article/10.3390/toxics14050377/s1, Table S1: The Limit of detection (LOD) of metal by ICP-MS; Table S2: Metal concentrations in different size fractions; Table S3: Summary of statistical parameters for metals (µg/m^3^) in halls by particle size.
