# Supplementary material for: Size Distribution and Metal Concentrations of Particulate Matter (PM) Sourced from Fireworks Shows in the Indoor Air of Wedding Halls in Bursa, Türkiye
Source: Toxics. 2026 Apr 28;14(5):377. doi: 10.3390/toxics14050377 (PMC13211617; doi:10.3390/toxics14050377)
Supplement: Supplementary file 1 [file toxics-14-00377-s001.zip › toxics-4198406-supplementary.pdf]

Supplementary

Table S1. The Limit of detection (LOD) of metal by ICP-MS.

Metals	LOD (ppb)
Fe	2.950
Ti	0.100
Sn	0.100
Al	2.420
Sr	0.150
Sb	0.070
Zn	6.970

Table S2. Metal concentrations in different size fractions.

Sample Number	*PM (μm)*	*Fe (μg/m³)*	*Ti (μg/m³)*	*Sn (μg/m³)*	*Al (μg/m³)*	*Sr (μg/m³)*	*Sb (μg/m³)*	*Zn (μg/m³)*
	PM>2.5	16.600	0.001	0.225	1.038	0.000	0.005	0.030
	1.0-2.5	0.100	0.001	0.180	3.428	0.001	0.010	0.052
Hall 1	***0.5-1.0***	12.500	0.001	0.915	1.100	0.020	0.000	0.040
	0.25-0.5	23.570	0.001	1.210	3.260	0.015	0.000	0.060
	PM<0.25	17.150	0.002	0.010	0.952	0.005	0.001	0.440
	PM>2.5	3.500	0.001	0.350	21.386	0.201	0.002	0.020
	1.0-2.5	0.100	0.001	0.480	32.562	0.286	0.003	0.136
Hall 2	***0.5-1.0***	4.250	0.001	0.780	43.380	0.375	0.004	0.472
	0.25-0.5	12.850	0.001	1.250	82.894	0.640	0.034	5.104
	PM<0.25	33.500	0.002	2.290	66.000	0.500	0.023	0.412
	PM>2.5	0.100	0.001	2.000	14.211	0.147	0.000	2.428
	1.0-2.5	39.100	0.001	0.320	90.454	0.738	0.012	6.164
Hall 3	***0.5-1.0***	4.900	0.001	0.600	179.306	0.513	0.008	32.530
	0.25-0.5	38.150	0.001	0.430	253.954	0.476	0.007	54.308
	PM<0.25	28.300	0.002	0.825	226.371	0.478	0.000	51.092
	PM>2.5	15.600	0.001	0.815	570.634	0.714	0.017	130.052
	1.0-2.5	14.850	0.001	0.900	799.328	0.863	0.018	145.702
Hall 4	***0.5-1.0***	0.150	0.001	0.605	222.291	0.237	0.005	41.638
	0.25-0.5	31.300	0.001	0.760	1029.660	1.352	0.039	375.411
	PM<0.25	30.900	0.002	1.115	893.083	1.574	0.033	181.193
	PM>2.5	81.600	0.01	1.265	956.288	1.607	0.145	43.642
	1.0-2.5	52.750	0	1.225	786.900	1.375	0.152	48.122
Hall 5	***0.5-1.0***	60.450	0	1.115	1115.572	1.471	0.161	47.913
	0.25-0.5	73.650	0	1.160	1110.257	1.441	0.159	40.451
	PM<0.25	58.500	0.001	1.555	836.340	1.290	0.139	36.120
	PM>2.5	51.500	0.005	0.900	776.537	1.414	0.149	42.891
	1.0-2.5	58.950	0	0.785	930.343	1.481	0.168	48.897
Hall 6	***0.5-1.0***	87.950	0.005	1.270	1596.532	2.098	0.228	63.378
	0.25-0.5	56.550	0	0.970	1094.828	1.471	0.164	45.917
	PM<0.25	49.450	0	1.375	1012.183	1.363	0.139	39.568
	PM>2.5	53.150	0.005	1.315	969.608	1.389	0.153	43.517
	1.0-2.5	60.700	0	1.200	1006.492	1.512	0.170	48.549
Hall 7	***0.5-1.0***	61.900	0	1.205	1170.274	1.540	0.171	49.294
	0.25-0.5	63.750	0	1.070	1161.566	1.534	0.166	49.577
	PM<0.25	46.600	0.001	0.890	999.840	1.387	0.145	42.684
	PM>2.5	55.150	0.005	1.185	1015.320	1.388	0.146	44.060
	1.0-2.5	63.850	0.005	1.005	1155.506	1.509	0.163	50.270
Hall 8	***0.5-1.0***	65.850	0	1.355	982.603	1.559	0.168	51.665
	0.25-0.5	63.950	0	1.350	1187.237	1.672	0.159	49.538
	PM<0.25	53.050	0.001	1.240	1111.877	1.617	0.154	47.032
	PM>2.5	55.400	0.010	1.765	987.240	1.483	0.138	41.943
	1.0-2.5	59.100	0	1.605	1077.394	1.581	0.157	45.003
Hall 9	***0.5-1.0***	68.950	0.005	1.415	1132.234	1.827	0.167	51.552
	0.25-0.5	63.450	0	2.320	1027.972	1.594	0.138	41.327
	PM<0.25	53.500	0	1.180	640.586	1.572	0.138	42.822
	PM>2.5	0.100	0.001	0.100	428.600	0.100	0.010	42.086
	1.0-2.5	77.850	0	1.210	1091.357	1.661	0.153	46.840
Hall 10	***0.5-1.0***	76.350	0.005	1.325	889.963	1.873	0.187	56.300
	0.25-0.5	67.900	0	1.635	1079.666	1.622	0.151	45.434
	PM<0.25	30.300	0	1.190	670.972	1.196	0.112	33.115

Table S3. Summary of statistical parameters for metals (µg/m³) in halls by particle size.

Metal	*Size Bin*	*Mean_value*	*Std_value*	*Min_value*	*Max_value*
Al	<0.25	88.97	56.72	0.95	226.37
Al	0.25-0.5	102.39	61.56	3.26	253.95
Al	0.5-1.0	78.12	56.53	1.10	179.31
Al	1.0-2.5	75.54	33.50	3.43	109.00
Al	>2.5	58.74	38.27	1.04	101.40
Fe	<0.25	40.13	13.77	17.15	58.50
Fe	0.25-0.5	49.51	21.22	12.85	73.65
Fe	0.5-1.0	44.33	34.47	0.15	87.95
Fe	1.0-2.5	42.74	28.01	0.10	77.85
Fe	>2.5	33.27	29.27	0.10	81.60
Sb	<0.25	0.09	0.07	0	0.15
Sb	0.25-0.5	0.10	0.07	0	0.17
Sb	0.5-1.0	0.11	0.09	0	0.23
Sb	1.0-2.5	0.10	0.08	0	0.17
Sb	>2.5	0.08	0.07	0	0.15
Sn	<0.25	1.17	0.58	0.01	2.29
Sn	0.25-0.5	1.22	0.51	0.43	2.32
Sn	0.5-1.0	1.06	0.31	0.60	1.41
Sn	1.0-2.5	0.89	0.45	0.18	1.60
Sn	>2.5	0.99	0.64	0.10	2.0
Sr	<0.25	1.10	0.56	0.01	1.62
Sr	0.25-0.5	1.18	0.58	0.02	1.67
Sr	0.5-1.0	1.15	0.78	0.02	2.10
Sr	1.0-2.5	1.10	0.59	0.00	1.66
Sr	>2.5	0.84	0.67	0.00	1.61
Ti	<0.25	0.85	0.75	0	2.00
Ti	0.25-0.5	0.35	0.47	0	1.00
Ti	0.5-1.0	1.90	2.18	0	5.00
Ti	1.0-2.5	0.80	1.53	0	5.00
Ti	>2.5	3.90	3.75	0.50	10.00
Zn	<0.25	47.45	50.29	0.41	181.19
Zn	0.25-0.5	70.71	108.70	0.06	375.41
Zn	0.5-1.0	39.48	22.23	0.04	63.38
Zn	1.0-2.5	43.97	41.82	0.05	145.70
Zn	>2.5	39.07	37.66	0.02	130.05
